# A Concise Method to Predict the Mean Dynamic Pressure on a Plunge Pool Slab

**DOI:** 10.3390/e24010045

**Published:** 2021-12-27

**Authors:** Maolin Zhou, Xin Li, Jianmin Zhang, Weilin Xu

**Affiliations:** State Key Laboratory of Hydraulics and Mountain River Engineering, Sichuan University, Chengdu 610065, China; zhoumaolin@scu.edu.cn (M.Z.); bixizhen@foxmail.com (X.L.); xuwl@scu.edu.cn (W.X.)

**Keywords:** dimensional analysis, parabolic trajectory, flip bucket, dynamic pressure, jet trajectory, plunge pool

## Abstract

Hydrodynamic pressure exerted on a plunge pool slab by jet impingement is of high interest in high dam projects. The present study experimentally investigated the characteristics of pressure induced by a jet through a constant width flip bucket (CFB) and a slit flip bucket (SFB). A pressurized plane pipe was employed in the flume experiments to control the inlet velocities in the flip buckets. A concise method is proposed to predict the mean dynamic pressure field. Its implementation is summarized as follows: First, the position of the pressure field is determined by the trajectories of free jets, and to calculate its trajectories, an equation based on parabolic trajectory theory is used; second, the maximum mean dynamic pressure is obtained through dimensional analysis, and then the pressure field is established by applying the law of Gaussian distribution. Those steps are integrated into a concise computing procedure by using some easy-to-obtain parameters. Some key parameters, such as takeoff velocity coefficient, takeoff angle coefficient, and the parameter $k_{2}$, are also investigated in this paper. The formulas of these coefficients are obtained by fitting the experimental data. Using the proposed method, the easy-to-obtain geometric parameters and initial hydraulic conditions can be used to calculate the maximum mean dynamic pressure on the slab. A comparison between experimental data and calculated results confirmed the practicability of this model. These research results provide a reference for hydraulic applications.

## 1. Introduction

**Figure 1:**
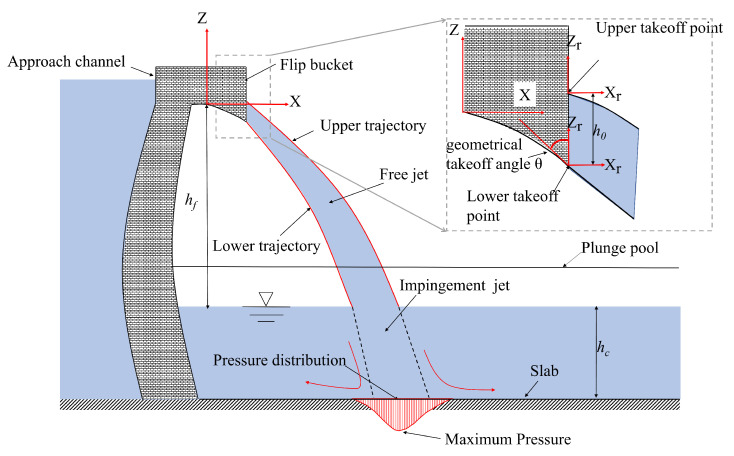
Scheme of the overflow discharge spillway and plunge pool.

Hydropower projects commonly utilize flip buckets to transfer stormwater from high elevations to plunge pools, e.g., the Mossyrock Dam in Washington State, USA [1], and the Jinping-I arch Dam in Sichuan, China [2]. There are two common types of flip buckets: constant width flip bucket (CFB) and slit flip bucket (SFB). Recently, a new type of flip bucket structure with a leak slab at the bottom has been proposed by Deng [3]. The present research focused primarily on CFB and SFB. Water released from a flip bucket outlet is directed downstream to produce a free jet [4]; however, an impingement jet is produced when a free jet plunges into a pool of water, as shown in Figure 1. Impingement jet impact on plunge pool slabs plays an important role in scouring a river bed due to its dynamic pressure [5]. Therefore, a thorough understanding and accurate prediction of the dynamic pressure on plunge pool slabs are essential in the design of high-elevation dams. The shapes of free jets are determined by geometrical characteristics of the flip buckets. Impingement jets have different properties depending on the shape of free jets, and impingement jets exert a dynamic pressure on the slab of the plunge pool. Their relationships are significant for engineering design in high-elevation dam projects. However, relevant research works on their relationships are very rare to this day. It is expected that for predicting dynamic pressure on plunge pool slabs, two particular aspects should be carefully considered: (1) location of the dynamic pressure field, and (2) maximum mean dynamic pressure and its related pressure distribution.

The location of the dynamic pressure is mainly determined by trajectories of free jets [6]. A free jet falling in still air has been extensively studied, taking into account air entrainment, trajectory, flow pattern, transverse velocity distribution, and energy dissipation [4,7,8,9,10]. Formulas derived from mass point dynamics were introduced to estimate jet trajectories [7,11]. The takeoff angles and takeoff velocities of a jet are needed in computing the jet trajectories. In general, prototype jet trajectories often deviate from the commonly adopted trajectory parabola proposed by the US Bureau of Reclamation [11]. One of the challenges of applying the parabolic trajectory is to obtain the takeoff angles of jets. Researchers pointed out that lower and upper jet trajectory takeoff angles are frequently different from the deflection angle of bucket slabs [12,13]. To redress the discrepancy between the takeoff angle and geometric takeoff angle, some empirical equations were presented to describe their relationship [14,15]. Wu and Xu [16] demonstrated that the takeoff velocity has a much larger effect on the impact point than the takeoff angle. Thus, another challenge is to determine the takeoff velocity at the takeoff point, which may vary with the water depth and geometric takeoff angle.

In plunge pool slabs, stability is determined by the highest mean dynamic pressure and its distribution, as the standard deviation of the distribution is small [5]. Water cushions with effective water pressures and water cushions without effective water pressures have been differentiated based on the pressures caused by the different situations in dynamics [17,18]. An initial study of dynamic pressure on plunge pool slabs began in the 1960s, and a formula integrating the momentum conservation law was set forth by Cola [19] to estimate the maximum mean dynamic pressure. The model consisted of four parameters: Water velocity when it reaches the water cushion, water cushion thickness, jet height, and an experimental coefficient. In terms of effective water cushions, the expression had proven useful [18]. As discussed by Guo [20], the air content of jets is a factor in maximum mean dynamic pressure and the distribution of pressure at the slab, and it was found that the maximum mean dynamic pressure decreases and the pressure distribution region become larger when air is entrained into jets. The typical pressure distribution was observed to obey the Gaussian distribution law well [21,22,23]. Some formulas were proposed to calculated mean or fluctuating dynamic pressure value and its distribution [24,25,26,27,28,29]. However, the required parameters used to calculate the pressure in the above literature are related to the cross-section of jets at the water cushion surface, including the shape of the cross-section, jet width, and entry velocity. It is an inconvenience for engineers to apply formulas because it is hard to obtain the aforementioned required parameters.

This paper presents a concise and convenient method for predicting the mean dynamic pressure field and its location on the plunge pool slab. In the present method, it is possible to evaluate the free jet trajectory in still air and a water cushion, the position of the jet’s falling point on the slab, the maximum pressure value caused by the jet, and the pattern of pressure distribution using parameters easily obtained from flip buckets. In addition to that, it is also a systematically based method for writing programs and quickly performing calculations. The method is unique as it correlates the dynamic pressure with the easy-to-obtain parameters, such as flow rate *Q*, the width of the flip bucket $b_{0}$, the depth of the water cushion $h_{c}$, and the geometrical takeoff angle of the flip bucket θ. To support the theoretical analysis, a physical model of an over-topping flow flip bucket and plunge pool are employed, and detailed measurements on trajectories of free jets and mean dynamic pressure on plunge pool slab were conducted.

## 2. Experimental Setup

**Figure 2:**
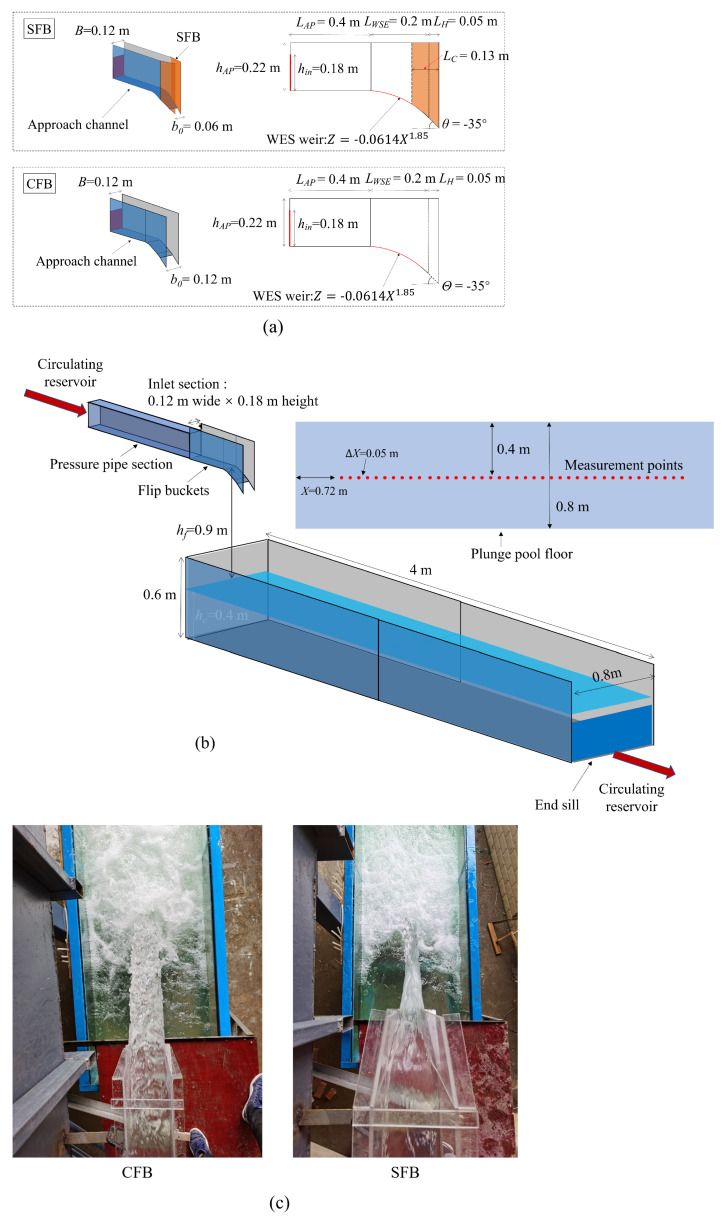
(**a**) The geometrical parameters of SFB and CFB. (**b**) Experimental model. (**c**) Flow type of the jet in still air.

A lab experiment was conducted to test a free jet launched from a flip bucket that hits the water cushion in a plunge pool. Figure 2 illustrates the experimental setups. The physical model consists of an approach channel with dimensions of 0.4 m long × 0.12 m wide × 0.25 m high, a flip bucket, a rectangular plunge pool with dimensions of 4.0 m long × 0.80 m wide × 0.65 m high, and a circulating reservoir. The approach channel and the flip bucket were made of Plexiglas; the plunge pool was made of glass. Water was supplied through the approach channel connected to a circulating water supply system. By using a thin plate weir, the discharge of water was measured to the nearest 0.01 L/s, and the flow ranged from 18.58 to 43.76 L/s. Two types of classic flip buckets were tested, a constant width flip bucket (CFB) and a slit flip bucket (SFB). Both types of buckets were connected to the approach channel by a waterways experiment station (WES) weir. As shown in Figure 1, the slab of the WES weir is determined by $Z=-0.0614X^{1.85}$, where X and Z are corresponding values in the X–Y coordinate system. The bottom slab of all flip buckets is retracted 0.05 m from the exits. The geometric takeoff angle ($\theta_{0}$) for both types of flip buckets is −35°. The exit width of the SFB shrinks evenly from 0.12 to 0.06 m within a length of 0.13 m, while the exit width of the CFB remains 0.12 m. The difference in height between the approach channel bottom and the plunge pool slab is 1.30 m.

The depth of the water cushion ($h_{c}$) was settled to 0.40 m by operating an adjustable baffle at the outlet of the plunge pool, which is considered an effective water cushion according to Puertas and Dolz’s research [18]. Figure 2b shows forty pressure sensors placed along the central line of the plunge pool slab to measure the time-average pressure (ΔP). The pressure sensor used in the experiment is the CY200 pressure sensor made by Chengdu Test Co., Ltd. This pressure signal is picked up and processed by a piezoresistive silicon crystal as the sensitive part. It then performs filtering, amplifies correction functions, and directly outputs the displayed and stored data. The sensor is connected to the hub via the connection line, and the hub is connected to the PC terminal via the network cable. It is possible to connect up to 20 CY200s at once to a hub. Samples are taken at 100 Hz, the total duration of sampling is 120 s, and the accuracy exceeds 0.01 kpa. The plotted data are the average of the measured instantaneous data. The height of the free jet at the takeoff cross-section was measured with a steel ruler ($h_{0}$).

A digital camera fixed on one side of the experimental system was used to take pictures of the free jet. Instantaneous images were used to obtain trajectory data. This is a non-intrusive measurement technique that has been proven useful by other researchers [30,31]. For the first step, a calibration board with 11 horizontal and 10 vertical uniform black and white square meshes was used to correct distortions. Next, all images of jets were greyed and binarized with thresholds measured at the air–water interface, after which an edge for representing the trajectory was determined. Dimension is converted from one image unit (pixel) to another by scaling the height of the flip buckets in the physical model.

Table 1 summarizes the range of the experimental parameters. The takeoff bulk velocity at the takeoff cross-section $\bar{U_{0}}$ is defined by $Q/(h_{0}\times b_{0})$, where *Q* = flow rate and $h_{0}\times b_{0}$ = area of the takeoff cross-section. The Froude number varies from 1.14 to 2.70 in the approach channel of the present experimental model, while the Reynolds number varies from 172,575 to 407,358. Instead of focusing on a specific high dam project, the article abstracts problems of overflow flood discharge and conducts laboratory experiments. When testing the hydraulics of a high dam project, the physical prototype must be scaled to the model size properly to obtain accurate results. The present study will focus on the distribution of mean dynamic pressure on the plunge pool slab, the typical jet trajectory of free jet flow, which satisfies the Froude criterion. According to the Froude similitude law, the values can be extrapolated from laboratory results for use in real engineering. However, scale effects may still will appear in a physical model because the air–water properties in supercritical flows cannot be discounted in terms of surface tension and viscosity effects in high-speed air–water flows. These effects can be minimized or accounted for with careful selection of the model size and interpretation of the results. In order to minimize this effect in vertical plunging jets, the head of the flow should be larger than 0.045 m [32,33]. According to Pfister and Hager [9], the amount of stable drop size is constant for both the primary and secondary jet disintegration. The water drops are supposed to be stable if W<10. The effect is mainly confined to the part of air concentrations over 90%, which is insignificant to define a jet surface. The maximum Weber number $W=(\rho U^{2}h_{in})/\sigma$ in the present experiment was below 11, where σ = water surface tension. In the present study, these limitations were respected. Hence, the trajectories of free jets are believed to not be affected by the width in the transversal direction (12 cm).

According to Figure 2c, the width of the free jet released by CFB remains constant while the width of the free jet released by SFB rapidly shrinks in the *Y* direction (transverse), and then remains small until the free jet jumps into the pool. Thus, a two-dimensional treatment of the flow motion is reasonable. Particular attention is paid to the distribution of pressure on the plunge pool floor in the *X* direction (streamwise direction) because it more directly determines the cost of the plunge pool floor.

## 3. Calculating the Trajectory of the Free Jet

### 3.1. General Equation

Various studies [6,7] indicated that both the lower and upper free jet trajectories can be approximated with the parabolic equation, based on the mass point dynamics. Takeoff points were defined as the origin of the $X_{r}-Z_{r}$ coordinate system (Figure 2). The $X_{r}-Z_{r}$ coordinate system is transformed from the X−Z coordinate system using Equations (1) and (2):

(1) $$\begin{array}{c} Z=\left\{\begin{array}{c} Z_{r}+Z_{0l}+h_{0}\;\mathrm{at}\;\mathrm{upper}\;\mathrm{edge}\\ Z_{r}+Z_{0l}\;\mathrm{at}\;\mathrm{lower}\;\mathrm{edge} \end{array}\right. \end{array}$$

(2) $$X=X_{r}+L_{WES}$$

where $Z_{0l}$ represents the coordinate value of lower takeoff points in the Z− axis of the X−Z coordinate system; $h_{0}$ represents the height of the free jet at the takeoff cross-section; $L_{WSE}$ represents the horizontal length of the WES weir.

The upper edge or lower edge of free jets can be calculated by the effective takeoff angle ($\theta_{0}$) and takeoff velocity ($U_{0}$). The equation of motion in a plane is:

(3) $$Z_{r}=X_{r}\tan\theta_{0}-\frac{gX_{r}^{2}}{2U_{0}^{2}\cos^{2}\theta_{0}}$$

where $X_{r}$ and $Z_{r}$ are the coordinates of the trajectory of the edge of free jets in the $X_{r}-Z_{r}$ coordinate system; *g* is the acceleration due to gravity; if a jet issues upward, $\theta_{0}$ is positive, and otherwise negative. The major obstacle of applying Equation (3) is to obtain the effective takeoff angle ($\theta_{0}$) and the takeoff velocity ($U_{0}$). It is necessary to replace these two parameters with some easy-to-obtain parameters. In the present study, some coefficients are introduced to calculate these parameters. The coefficients introduced in this paper are all obtained by fitting the experimental results.

### 3.2. Mean Velocity Coefficients

**Figure 3:**
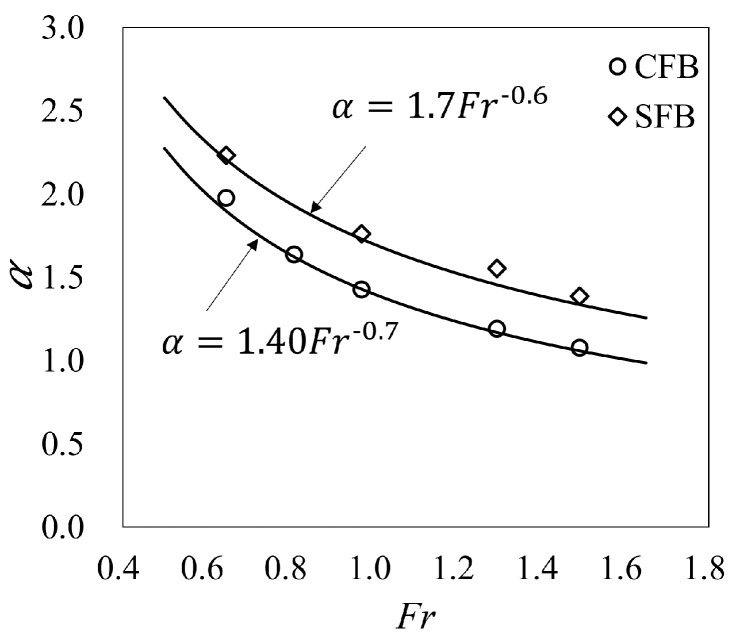
Mean velocity coefficient at the free jets takeoff cross-section (CFB and SFB).

To make the application of the trajectory in the case of dam over-topping flow as convenient as possible, the trajectory is expressed as a function of the inlet velocity (*U*). The velocity of jet flow from a flip bucket can be easily related to the reservoir head. The relationship of the inlet velocity *U* at the approach channel and the mean takeoff velocity ($\bar{U_{0}}$) at the takeoff cross-section is:

(4) $${\bar{U}}_{0}=\alpha U$$

where α is the mean velocity coefficient. Figure 3 shows that for a certain flip bucket, and α gradually decreases with the increase in Froude number ($Fr=U/\sqrt[{}]{gh_{in}}$). The mean velocity coefficients $\alpha_{CFB}$ and $\alpha_{SFB}$ are given by functions of *Fr* as:

(5) $$\alpha_{CFB}=1.4Fr^{-0.7}$$

(6) $$\alpha_{SFB}=1.7Fr^{-0.6}$$

### 3.3. Takeoff Velocity Coefficients

**Figure 4:**
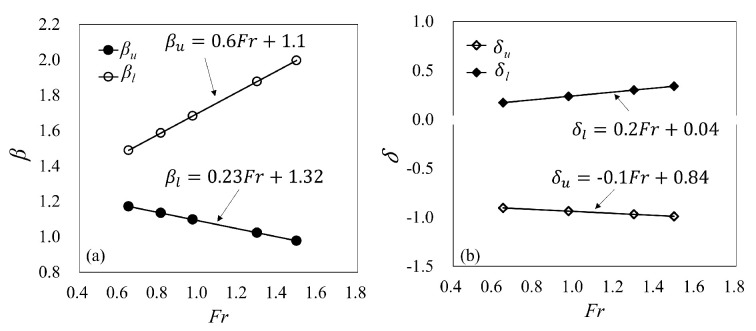
(**a**) Velocity coefficient at upper and lower takeoff points (CFB). (**b**) Takeoff angle coefficient at upper and lower takeoff points.

Note that the takeoff velocity at the upper (or lower) takeoff point may differ from the takeoff bulk velocity ($\bar{U_{0}}$). The relationship of the $U_{0u}$ (or $U_{0l}$) and ($\bar{U_{0}}$) can be expressed as:

(7) $$U_{0u}=\beta_{u}\bar{U_{0}}$$

(8) $$U_{0l}=\beta_{l}\bar{U_{0}}$$

where $\beta_{u}$ and $\beta_{l}$ are the upper and lower takeoff velocity coefficients, respectively. The measured trajectories were used as benchmark data, and the values of takeoff velocity coefficients were adjusted accordingly. For CFB, these takeoff velocities are larger than the mean takeoff velocities ($\bar{U_{0}}$) and $U_{0u}$ gradually increases with the increase in Fr, while $U_{0l}$ decreases with the increase in Fr, as shown in Figure 4a. For SFB, these takeoff velocities are identical to the mean takeoff velocities ($\bar{U_{0}}$) since the height of the free jet at the takeoff cross-section ($h_{0}$) is small. The values of takeoff velocity coefficients can be given as follows.

For CFB:

(9) $$\beta_{u}=0.6Fr+1.10$$

(10) $$\beta_{l}=-0.23Fr+1.32$$

For SFB:

(11) $$\beta_{u}=\beta_{l}=1$$

### 3.4. Takeoff Angle Coefficients

Various studies indicate that using the geometric takeoff angle (θ) for trajectory computation may result in unacceptable errors [9]. Consequently, the pressure field position on the plunge pool slab is sometimes closer than estimated with θ. The relationship of the effective takeoff angle at the upper ($\theta_{0u}$) and the lower ($\theta_{0l}$) takeoff points and the geometric takeoff angle (θ) can be expressed as:

(12) $$\theta_{0u}=\delta_{u}\theta ;\theta_{0l}=\delta_{l}\theta ;$$

where $\delta_{u}$, $\delta_{l}$ represent the takeoff angle coefficient at the upper and the lower takeoff points, respectively. The variables $\delta_{u}$ and $\delta_{l}$ were used to fit the experimental trajectory data to Equation (3). For CFB, the virtual angles ( $\theta_{0u}$ and $\theta_{0l}$) at both the upper and the lower takeoff points are identical to the geometrical angles. For SFB, these virtual angles at the upper takeoff points ($\theta_{0u}$) are mostly significantly larger than the geometrical angles (θ), while the virtual angles at the lower takeoff points ($\theta_{0l}$) are smaller than the geometrical angles (θ), as shown in Figure 4b. Note again that the geometrical angle (θ) in the present research is fixed at −35°. For all data sets, the takeoff angle coefficient is as follows.

For CFB:

(13) $$\delta_{u}=\delta_{l}=1$$

For SFB:

(14) $$\delta_{u}=-0.1Fr+0.84$$

(15) $$\delta_{l}=0.2Fr+0.04$$

**Figure 5:**
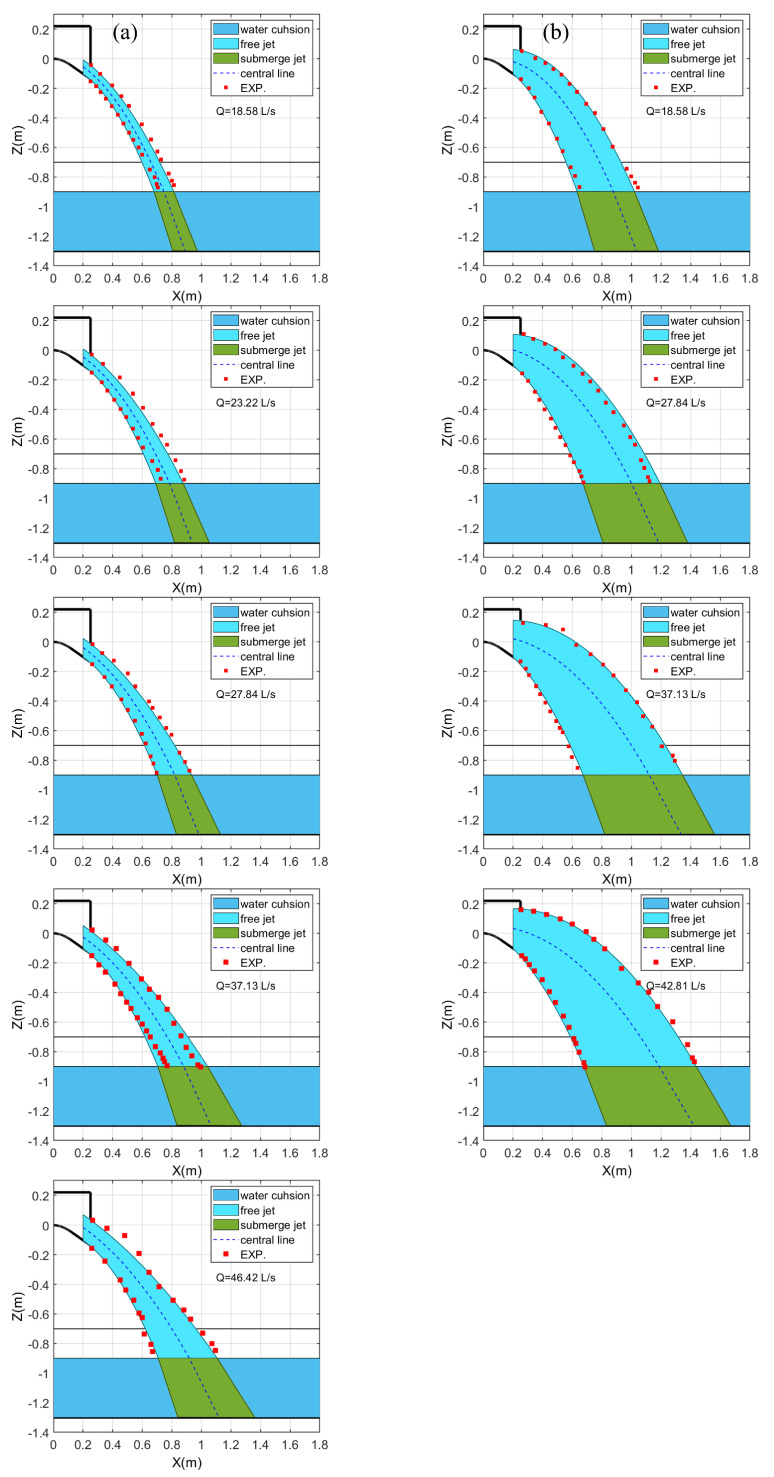
Comparison of the measured data from EXP. with the trajectory computed by using Equation (3): (**a**) CFB, (**b**) SFB.

Figure 5 shows a comparison of data sets predicted by using Equation (3). This result indicates that free jets issued from CFB and SFB can be well estimated by Equation (3).

## 4. Predicting the Dynamic Pressure

### 4.1. Location of the Stagnation Point

The central point of the impact area of the dynamic pressure due to the inclined submerged jet is the stagnation point where the maximum mean dynamic pressure on the slab occurred. It is assumed that the position of the stagnation point can be obtained by calculating the trajectory of the mass point at the center of the takeoff cross-section. The trajectory is called the central line in the present paper. The central line can be divided into two parts: a free jet section and an impingement jet section. In the free jet section, the central line can be calculated by Equation (3). The coordinate value needs to be adjusted using Equations (16) and (17):

(16) $$Z=Z_{r}+Z_{0l}+h_{0}/2$$

(17) $$X=X_{r}+L_{WSE}$$

The takeoff velocity coefficient ($\beta_{c}$) and the takeoff angle coefficient ($\delta_{c}$) of the central line can be obtained as:

(18) $$\beta_{0c}=\left(\beta_{0u}+\beta_{0l}\right)/2$$

(19) $$\delta_{0c}=\left(\delta_{0u}+\delta_{0l}\right)/2$$

In the impingement jet section, the central line can be approximated to a straight line tangential to the curvy central line upon entering the water cushion [34]. Figure 5 shows the comparison of the jet profile of the experiment and the one predicted by the aforementioned methods. The digital camera fixed on the side of the experimental system was used to take pictures of the free jet, and the profile data of the free jet was obtained through digital graphics processing. The predicted profiles of free jets and impingement jets are plotted along with central lines. The data calculated by the aforementioned methods agree well with the experimental data. Meanwhile, the end point of the central line is the stagnation point, denoted as $X_{s}$.

### 4.2. Maximum Mean Dynamic Pressure

The mean dynamic pressure, denoted as ΔP, is defined as the time-average pressure (the pressure must be divided by the water-specific weight, in order to obtain a height) on the plunge pool slab minus the average water depth in the plunge pool. ΔP is caused by the remaining momentum of the impingement jet. According to the theory of jets, the velocity attenuation is influenced by the length of the potential core, which is significantly determined by the characteristic width of the impingement jet (φ) [35]. In some literature, the characteristic width refers to the width or diameter of impingement jets [18,20,29]. Therefore, the accurate estimation of characteristic width (φ) of impingement jets is an essential step to calculate the value of dynamic pressure. As shown in Figure 5, there are huge differences in the initial cross-section shape of impingement jets resulting from different flip buckets. The initial cross-section shape is wide in the lateral-wise direction and relatively narrow in the streamwise direction when the free jet is discharged from the CFB. Hence the characteristic width (φ) is considered dependent on the initial height ($h_{0}$) of free jets. The initial cross-section shape is much narrower in the lateral-wise direction and prolonged in the streamwise direction when the free jet issues from the SFB, and the characteristic width (φ) is determined by the initial width ($b_{0}$) of free jets. Thus, for CFB, $\varphi =h_{0}$, and for SFB, $\varphi =b_{0}$, as shown Table 2.

The basic assumption is that maximum dynamic pressure is related to the initial velocity of the impingement jet ($U_{0}^{2}$), the characteristic width (φ), the depth of the water cushion ($h_{c}$), and water density (ρ), described by:

(20) $$\Delta P_{\max}=f\left(U_{0}^{\prime },\varphi ,h_{c},\rho \right)$$

The following formula can be obtained by dimensional analysis:

(21) $$\Delta P_{\max}=f\left(\frac{\varphi }{h_{c}}\right)U_{0}^{\prime 2}\rho$$

where $U_{0}^{\prime 2}$ is related to inlet velocity (*U*) and the height difference ($h_{f}$) between the approach channel’s bottom and the water cushion, as given below:

(22) $$U_{0}^{\prime 2}=U^{2}+2gh_{f}$$

Equation (21) can be rewritten as:

(23) $$\frac{\Delta P_{\max}}{\left(U^{2}+2gh_{f}\right)\rho }=f\left(\frac{\varphi }{h_{c}}\right)$$

**Figure 6:**
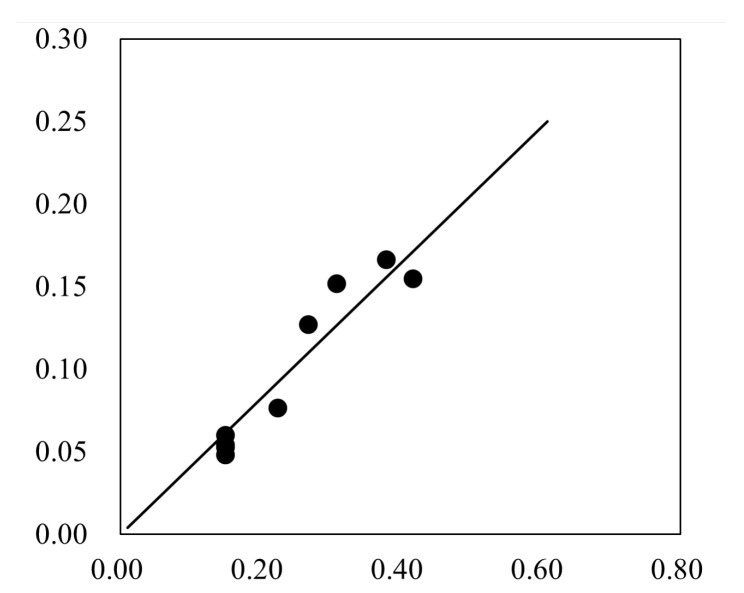
The relationship between $\Delta P_{\max}(U^{2}+2gh_{f})\rho$ and $\frac{\varphi }{h_{c}}$.

Figure 6 shows the relationship between $\Delta P_{\max}(U^{2}+2gh_{f})\rho$ and $\frac{\varphi }{h_{c}}$. Thus, we obtain:

(24) $$\Delta P_{\max}=0.41\rho \frac{\varphi }{h_{c}}\left(U^{2}+2gh_{f}\right)$$

### 4.3. Distribution of the Mean Dynamic Pressure

**Figure 7:**
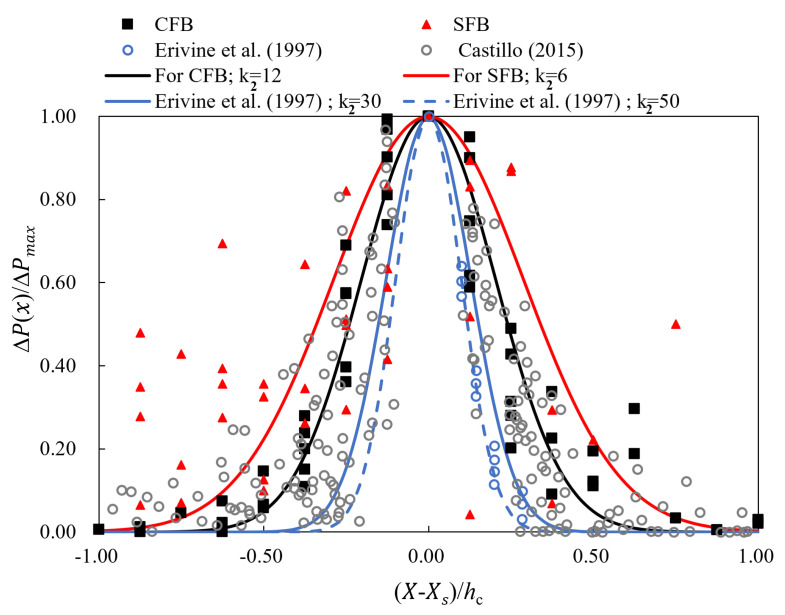
Distributions of non-dimensional mean dynamic pressure on the plunge pool slab.

Ervine et al. [25] showed how the mean dynamic pressure coefficient varies radially around the impingement jet. The distribution of the pressure coefficients can be thought of as the distribution of the pressure. The data revealed a general expression of the distribution:

(25) $$\frac{\Delta P(X)}{\Delta P_{max}}=e^{-k_{2}{\left(\frac{X-X_{s}}{h_{c}}\right)}^{2}}$$

where $X_{s}$ is the coordinate value of the stagnation point in the X−Z coordinate system, the horizontal distance to the stagnation point is $X-X_{s}$, and the water cushion depth is $h_{c}$. A non-dimensional distribution of the mean dynamic pressure is shown in Figure 7 in the streamwise direction on the plunge pool slab. For round jets, the parameter $k_{2}$ varied from 30 for shallow pools to 50 for deep pools [25]. The parameter $k_{2}$ varied for the rectangular jet case [36]. As for our experiment cases, CFB had a parameter of $k_{2}$ = 12, while SFB had a parameter of $k_{2}$ = 6. The onset of the jet breakup in still air was found to occur at a distance of approximately 100 times the diameter of the nozzle [37]. In the present study, $h_{f}$⁄φ = 5.39−15 shows that the jet flow does not break up in still air. It is important to note that this formula can only be used to predict the dynamic pressure field on the floor if there is an effective water cushion in the plunge pool.

## 5. Discussion

**Figure 8:**
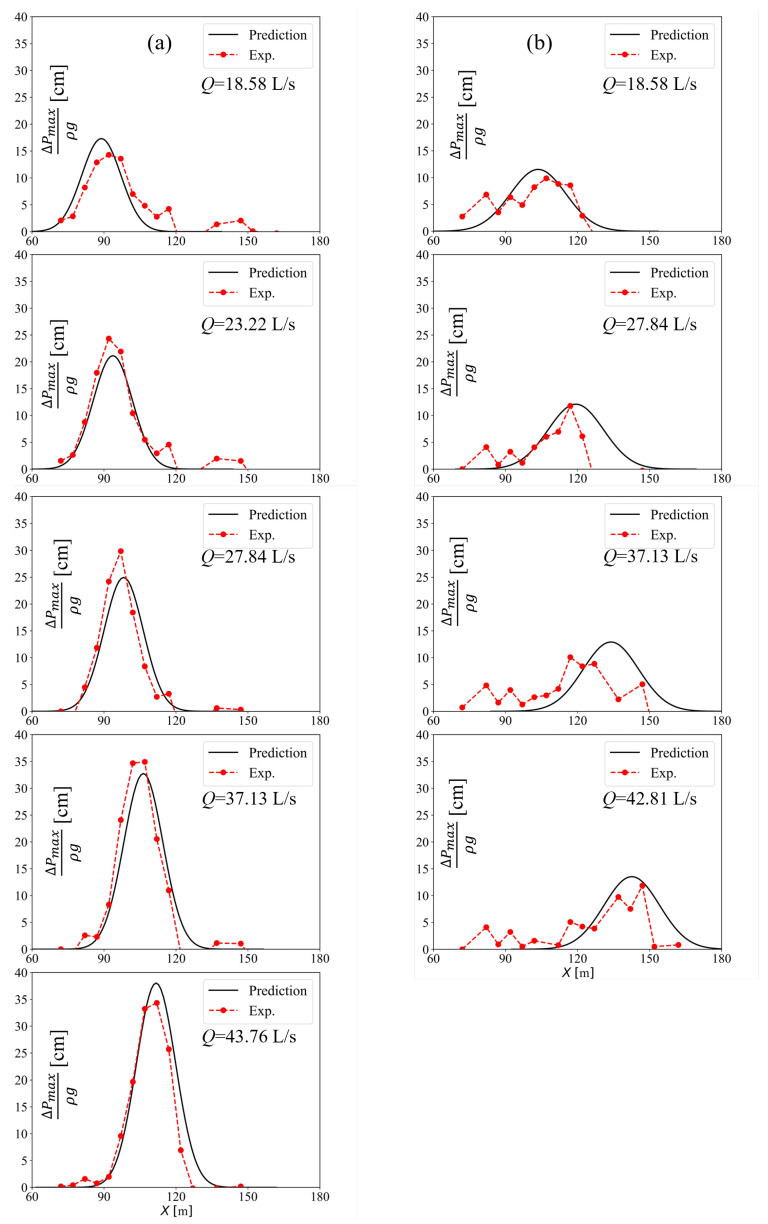
The comparison of the predicted mean dynamic pressure field with the corresponding experimental results: (**a**) CFB; (**b**) SFB.

In Table 3, the method used to predict the mean dynamic pressure of a slab is presented. The required parameters include *U*, Fr, $h_{0}$, $b_{0}$, θ, $h_{c}$, $h_{f}$, $\beta_{u}$, $\beta_{l}$, $\delta_{l}$, $\delta_{u}$, and $k_{2}$. The parameters of *U*, Fr, $h_{0}$, $b_{0}$, θ, $h_{c}$, and $h_{f}$ are easily obtained in actual engineering practice, while the parameters of α, $\beta_{u}$, $\beta_{l}$, $\delta_{l}$, $\delta_{u}$, and $k_{2}$ for CFB and SFB have been acquired by fitting experimental data. As illustrated in Figure 8, the predicted mean dynamic pressure field was compared with the corresponding experimental results. The experimental data were measured by pressure sensors that were placed on the central line of the plunge pool slab.In the CFB case, since the jet flow has a more concentrated cross-section, pressure distribution along the streamwise direction is more concentrated and its distribution is more Gaussian. There is little pressure variation on the slab because of the stable width of the jet flow. In the SFB case, the jet flow diffuses as the “—” type along the streamwise direction, so the range of pressure distribution is wider. Since the jet flow’s width fluctuates as more air enters the free jets [38], there is a large variation in pressure on the slab, so there is less agreement with predicted values and the pressure is more widely distributed in the *X* direction.

## 6. Conclusions

The prediction process of dynamic pressure on a plunge pool is of interest to the water supply and transfer systems. In the present study, the mean dynamic pressure was measured, and the effects of the inlet velocity and types of flip buckets were investigated using a physical hydraulic model. This study proposed a concise method using easy-to-obtain parameters to predict the mean dynamic pressure field on the plunge pool slab. In order to reach the goal, the convenient methods for predicting trajectories of free jets and the maximum mean dynamic pressure and related pressure distribution were presented. The following conclusions were developed:

1. The computation of the trajectory of a free jet should utilize both takeoff velocities ($U_{0u}$ and $U_{0l}$) and effective takeoff angles ($\theta_{0u}$ and $\theta_{0l}$) at the upper and the lower takeoff points (Equation (3)). The takeoff velocities can be estimated with reasonable accuracy using inlet velocity (*U*), the Froude number (Fr), and takeoff coefficients (β). The takeoff angles can be assessed by the geometrical takeoff angle (θ) and takeoff angle coefficients. Those coefficients for CFB and SFB were investigated in the present research.

2. The location of the stagnation point can be approximated to be the end point of the central line, which is the trajectory of the mass point at the center of the takeoff cross-section. An equation to calculate the maximum mean dynamic pressure was derived from dimensional analysis. The equation offered by Ervine [25] was used to predict the pressure distribution in the streamwise direction. The values of $k_{2}$ for CFB and SFB were 12 and 6, respectively.

## Figures and Tables

**Table 1:** Summary of experiments.

Test Number	Discharge *Q* (L/S)	Inlet Velocity *U* (m/s)	Type of the Flip Bucket
1	18.58	0.86	CFB
2	23.22	1.08	CFB
3	27.84	1.29	CFB
4	37.13	1.72	CFB
5	43.76	2.03	CFB
6	18.58	0.86	SFB
7	23.22	1.08	SFB
8	27.84	1.29	SFB
9	43.76	1.98	SFB

**Table 2:** The value of the characteristic width φ.

Type	*Q* (L/S)	$h_{0}$ (m)	$B_{0}$ (m)	φ (m)
CFB	18.58	0.090	0.120	0.090
CFB	23.22	0.108	0.120	0.108
CFB	27.84	0.124	0.120	0.124
CFB	37.13	0.152	0.120	0.152
CFB	43.76	0.167	0.120	0.167
SFB	18.58	0.090	0.060	0.060
SFB	23.22	0.108	0.060	0.060
SFB	27.84	0.124	0.060	0.060
SFB	42.81	0.167	0.060	0.060

**Table 3:** The steps used to predict the mean dynamic pressure on the plunge pool slab.

Step	Goal and Approach	Requisite Steps (Rs.) and Parameters (Rp.)
1	Goal: obtain the takeoff velocity ($\bar{U_{0}}$ ) at takeoff cross-section; Approaches: Equations (4)–(6).	Rs.: None Rp.: *U*, Fr, α
2	Goal: obtain takeoff velocities at upper and lower takeoff points $U_{0u}$ and $U_{0l}$; Approaches: Equations (7) and (8).	Rs.: None Rp.: $\bar{U_{0}}$, $\beta_{u}$, $\beta_{l}$
3	Goal: obtain effective takeoff angle at lower takeoff points ($\theta_{0u}$ and $\theta_{0l}$); Approach: Equation (12).	Rs.: None Rp.: θ, $\delta_{u}$, $\delta_{l}$
4	Goal: obtain the location of the stagnation point ($X_{s}$); Approaches: calculate the central line by Equations (3), (16) and (17), and the end point of central line is the stagnation point.	Rs.: step 1, 2, and 3 Rp.: θ, $\delta_{u}$, $\delta_{l}$
5	Goal: calculate the maximum mean dynamic pressure $\Delta P_{max}$; Approach: Equation (24).	Rs.: None Rp.: $h_{0}$, $b_{0}$, *U*, $h_{c}$, $k_{2}$
6	Goal: obtain the distribution of the dynamic pressure ΔP(X); Approach: Equation (25).	Rs.: step 4 and 5 Rp.: $X_{s}$, $\Delta P_{max}$, $h_{c}$, $k_{2}$

## Data Availability

Not applicable.

## Abbreviations

The following abbreviations are used in this manuscript:

*Q*	Flow rate
$\bar{U_{0}}$	Takeoff bulk velocity
*U*	Inlet velocity
$U_{0}$	Takeoff velocity
$U_{0u}$	Takeoff velocity at the upper takeoff point
$U_{0l}$	Takeoff velocity at the lower takeoff point
Fr	Froude number
$X_{s}$	Position of the stagnation point
$h_{0}$	Height of the free jet at the takeoff cross-section
$b_{0}$	Width of the flip bucket
$h_{c}$	Depth of water cushion
$h_{f}$	Height difference between approach channel bottom and water cushion
φ	Characteristic width of free jets
θ	Geometrical takeoff angle of the flip bucket
$\theta_{0}$	Effective takeoff angle
$\theta_{0u}$	Effective takeoff angle at the upper takeoff point
$\theta_{0l}$	Effective takeoff angle at the lower takeoff
α	Mean velocity coefficient
$\beta_{u}$	Upper takeoff velocity coefficients
$\beta_{l}$	Lower takeoff velocity coefficients
$\theta_{u}$	takeoff angle coefficient at the upper takeoff point
ΔP	Mean dynamic pressure
$\Delta P_{max}$	Maximum mean dynamic pressure
